# Mild traumatic brain injury/concussion and female sexuality, a scoping review of the literature

**DOI:** 10.1186/s40621-020-0232-9

**Published:** 2020-03-02

**Authors:** Martina Anto-Ocrah, Kimberly Tiffany, Linda Hasman, Edwin van Wijngaarden

**Affiliations:** 1 Department of Emergency Medicine, University of Rochester School of Medicine and Dentistry, Rochester, New York USA; 2 Department of Obstetrics and Gynecology, University of Rochester School of Medicine and Dentistry, Rochester, New York USA; 3 Department of Neurology, University of Rochester School of Medicine and Dentistry, Rochester, New York USA; 4 University of Rochester, Rochester, New York USA; 5 Miner Library, University of Rochester School of Medicine and Dentistry, Rochester, New York USA; 6 Department of Public Health Sciences, University of Rochester School of Medicine and Dentistry, Rochester, New York USA

**Keywords:** Traumatic brain injury (TBI), Concussion, Women, Female, Gender, Sexual function, Neurosexuality, Sexual health, Scoping review

## Abstract

**Background:**

The American Congress of Rehabilitation Medicine (ACRM) in 2010 called for more head injury research on gender disparities to bridge the gender gap for the short-and long-term effects of TBI, including sexual and reproductive outcomes. In this paper, we review the state of the literature before and after the ACRM announcement, and evaluate how research teams have considered females and mildly injured TBI(mTBI)/concussion groups in post-TBI-related changes in sexual functioning.

**Methods:**

The research question for this scoping review was “what is the state of the literature in the evaluation of post-TBI sexual changes for women, and individuals with mTBI?” Using the 2010 ACRM call for action as a line of demarcation, we compared our findings before and after the 2010 announcement.

**Results:**

We identified 9 research studies that addressed sexual functioning changes in females and mTBI/concussion groups. Four of the nine were published before the 2010 ACRM announcement, and five were published after. The representation of female research participants increased steadily over the 28-year timespan. The proportion of individuals with mTBI included in the post-2010 era was higher than the earlier time period. Consistently, women with mTBI reported more adverse sexual outcomes compared to male cohorts, orthopaedic, and non-injured comparison groups. This observation persisted regardless of recruitment site (rehabilitation center/Emergency Department (ED)/Community) or time of outcome assessment (acute versus chronic). The findings also remained despite the heterogeneity of survey questionnaires used to evaluate sexual functioning outcomes. Excluding the most recent 2019 study, none of the research groups compared the findings by TBI severity, making it difficult to fully understand how concussion-related sexual changes compare to more severe forms of the head injury. The long term impacts of the sexual changes, such as infertility and relationship discord were also absent across all studies; even though most evaluated outcomes chronically (some as far out as 20 years post injury).

**Conclusion:**

The number of publications in the era before the ACRM call for action and afterwards were almost identical. In order to tailor interventions for the appropriate groups of TBI patients, more neurosexuality research is needed to increase awareness of the importance of sexuality as a health outcome for individuals with neurodisabilities.

“At least since Aristotle, natural historians [have] given preference to the study of male bodies, or more precisely, the bodies of male citizens. Woman, considered a monstrous error of nature, was studied for her deviation from this male norm” ~Londa Schiebinger’s *Nature’s Body* (Schiebinger 2013*).*

## Introduction

Since the origins of science and medicine, the “woman” has been considered negligible. Historically, it was believed that the female form was simply a male ‘turned outside in’ (Schiebinger 2013). Anatomists argued that ovaries were female testicles and the uterus was the female scrotum. Scientists argued that because there weren’t any fundamental differences between males and females-other than physical size and reproductive function- medical education needed to be focused on the male as the “norm” (Schiebinger 2013; Criado 2019; Clayton 2018; Beery and Zucker 2011). This line of thinking has, over time, resulted in serious consequences for the medical treatment of women. In 1977, after the United States’ Food and Drug Administration (FDA) issued guidelines to exclude women of child bearing potential from drug trials (Criado 2019; Clayton 2018), the National Institutes of Health (NIH) provided evidence that several drugs had to be recalled because of their severe adverse effects in women, who had been under-represented in earlier drug trials. A 2009 review of the basic science literature revealed a male bias in 8 of the 10 fields evaluated, including neuroscience, physiology, pharmacology and endocrinology (Beery and Zucker 2011). In targeting immune disorders for pharmacological interventions, the absence of sex-based differences in study design and analysis has historically led to ‘one drug’ treatment regimens for both men and women” (Beery and Zucker 2011; Fish 2008). In 2005, 8 of 10 prescription drugs withdrawn in the U.S. were removed because of differences in side effects and health issues in women (Fish 2008).

The NIH has since mandated the consideration of “sex as a biological variable” (SABV) in all clinical research (Clayton 2018). Recognizing that biological sex-which includes both male and *female* bodies-affects cell physiology and function, symptoms and manifestations of disease, and responses to treatment, the initiative was created to ensure that women, who now account for roughly half of the US population, are considered in medicine and clinical research (National Institutes of Health 2018).

Human sexuality, according to the World Health Organization (WHO), is an essential aspect of health (World Health Organization 2006). Sexuality is central to survival, and enhances human lives (World Health Organization 2006; McAnulty and Burnette 2006). And although sexual response has requisite biological underpinnings, it is contextualized by a myriad of intrapersonal, interpersonal, and cultural factors (McAnulty and Burnette 2006; Association, A.P 2013). Poor sexual functioning-a pathology in the experience of sexual arousal, interest, desire and response-has been associated with adverse physical and mental health; and is a marker of quality of life (McAnulty and Burnette 2006; Association, A.P 2013; Nappi et al. 2016). Traumatic Brain Injury (TBI), often referred to as the “silent epidemic,” is one of the leading causes of death and disability globally (Centers for Disease Control and Prevention 2017; Dewan et al. 2018), and has often been associated with changes in sexual functioning (Latella et al. 2018; Moreno et al. 2013; Dyer and das Nair 2014; Moreno et al. 2012). In the US alone, the Centers for Disease Control and Prevention (CDC) estimates that over 2.8 million TBI-related Emergency Department (ED) visits, hospitalizations and deaths occur annually (Centers for Disease Control and Prevention 2017; Centers for Disease Control and Prevention 2016; Nelson et al. 2019), with rates in the female population increasing just as rapidly as in male cohorts. According to the most recently available data from the CDC (Centers for Disease Control and Prevention 2017; Centers for Disease Control and Prevention 2016) between 2007 to 2010, TBI rates among men grew from 491.6/100,000 to 800.4/100,000, a 63% increase. Similarly, female rates surged in the same time period, rising by 49% from 424.3/100,000 to 633.7/100,000 (Centers for Disease Control and Prevention 2017; Centers for Disease Control and Prevention 2016). While TBI-related injuries in men tend to be sports related, the primary injury mechanism for women is via motor vehicle crashes (Centers for Disease Control and Prevention 2017; Centers for Disease Control and Prevention, National Center for Injury Prevention and Control Division of Unintentional Injury Prevention REPORT TO CONGRESS 2015; Cassidy et al. 2014; Laker 2011). Women make up an increasing and substantial proportion of the TBI population.

Three out of every four TBIs is a *mild* traumatic brain injury (mTBI) or concussion, a bump, blow or jolt to the head that disrupts the normal function of the brain (Centers for Disease Control and Prevention 2016; Nelson et al. 2019). Traumatic forces imposed on the brain during the concussive injury can result in damages to several brain structures and functions, including the anterior pituitary gland (Schneider et al. 2007; West and Sharp 2014; Tanriverdi et al. 2010), the structure responsible for homeostatic regulation of sexual and reproductive functioning in humans. Damage to this essential organ could result in an injury-induced state of hypopituitarism, a condition where the pituitary gland fails to produce (adequate amounts of) one or more of its hormones, including follicle stimulating hormone (FSH) and luteinizing hormone (LH) (Schneider et al. 2007; West and Sharp 2014; Tanriverdi et al. 2010). Without proper regulation of FSH and LH, human reproduction-including sexuality, is impacted. In women, this would mean dysregulation of estrogen, progesterone and testosterone; the ovarian hormones that regulate sexual arousal, interest, desire and response for the female sex (McAnulty and Burnette 2006; Giacomo Ciocca et al. 2016; Cappelletti and Wallen 2016).

Neurosexuality is an emerging area of study and practice that focuses on the relationship between brain and sexual function in individuals with and without neurological disorders (Moreno et al. 2012; Moreno et al. 2017). Using a transdisciplinary framework, neurosexuality encourages researchers and providers to integrate the perspectives of natural, social, and health sciences in a humanities context to build a comprehensive understanding of the neural correlates of sexual behavior (Moreno et al. 2017). A number of research endeavors have evaluated sexual changes following TBI. Much of the literature, however, focuses on outcomes in men and/or those with moderate and severe TBI (Latella et al. 2018; Moreno et al. 2013; Grashow et al. 2019). In 2010, the American Congress of Rehabilitation Medicine (ACRM) issued a call for action (Harris et al. 2012), to reduce gender disparities in head injury research, and recommended that researchers and stakeholders bridge the gender gap for the short-and long-term effects of TBI, including sexual and reproductive outcomes. To promote the reduction of gender disparities in TBI research, the ACRM created the Girls and Women with Acquired Brain Injury Task Force (Colantonio 2016; Xie et al. 2019) to encourage researchers to apply a sex/gender framework to TBI research.

In this paper, we provide a scoping review of the literature to evaluate the existing body of research on female sexual functioning after TBI. Guided by the principles of neurosexuality, we compare the state of the literature before and after the ACRM announcement, and evaluate how research teams have considered injured females and concussion groups in evaluating TBI-related changes in sexual functioning.

## Methods

We chose a scoping review for this project to provide a preliminary overview of the existing gaps in the literature. Though both scoping reviews and systematic reviews use rigorous and transparent methods, the key differences between the two is in their differing purposes and aims (DiCenso et al. 2010). Scoping reviews seek to “map” all the relevant literature on a broad topic, with the goal of providing preliminary assessments of the extent, range and nature of research activity on the topic of interest. Systematic reviews on the other hand, are intended to sum up the best available research on a specific question and are less exploratory (DiCenso et al. 2010).

We followed the 5 steps recommended by Arksey and O’Malley (Pelaccia et al. 2019; De Allegri et al. 2018; Arksey and O'Malley 2005) in conducting this scoping review, as outlined.

### In step 1, we identified the research question

which was to evaluate the existing literature for publications that have included mTBI and women, in the evaluation of post-TBI sexual changes. Using the 2010 ACRM call for action as a line of demarcation, we stratified our findings to compare publications in the period before and including 2010, and those published in 2011 and after.

### In step 2, we identified relevant studies

Our search was designed to capture primary research that explored the topic of head injury and female sexual functioning in peer-review journals. With the assistance of a librarian (LH), the first author (MAO) initiated a comprehensive search of bibliographic databases (with citation tracking). We used the three largest and most comprehensive library databases for this purpose: Pubmed, Embase and Web of Science. Our librarian team member (LH) worked with MAO to select appropriate search terms (Appendix). The search was restricted to publications in the English language, that spanned 1980 to 2019. We used such a broad timespan to ensure that it would capture the earliest and most recent publication years relevant to the project. We conducted a series of pilot searches to ensure that the final search would retrieve three key articles (O’Carroll (1991) (O'Carroll et al. 1991), Hibbard (2000) (Hibbard et al. 2000a), Anto-Ocrah (2019) (Anto-Ocrah et al. 2019a)). The search terms were modified iteratively throughout the process, and the final search was performed in September 2019. To make sure that our search was capturing all available literature on the topic, we additionally searched the references of other published reviews of a similar nature to exhaust all search options.

Studies were extracted and managed in the EndNote bibliographical software (× 8.2 Copyright 2016 Clarivate Analytics) (EndNote X8 Clarivate Analytics 2016), using the following inclusion criteria:

1. primary research papers that
2. evaluate sexual functioning as the outcome
3. include females in the study population
4. include mTBI or concussion in the TBI group
5. focus on human participants, and not animal models

### In step 3, we selected studies to be included in the review

MAO reviewed the title and abstract of each article that was abstracted, and requested the full text article of those papers that met the inclusion criteria. After reviewing the full texts, MAO, with the supervision of EvW then decided on which studies to include in the final review. KT did a final review of the selected papers to ensure that they met the inclusion criteria before the records were charted in Step 4.

### In step 4, we charted the data

We used a table in microsoft word to organize and categorize relevant information from the studies retained for the review. To ease understanding by the reader, we created a number of categories to classify the information extracted. We identified the year of publication, authors and country. We also noted each study’s sample sizes (with delineation of injured and non-injured groups), proportion of female and male study participants, proportion of mTBI research participants, where patients were recruited from (the ED, in-hospital, in the community, etc), measures used to evaluate concussion-related sexual outcomes and the study design. We then grouped studies into those published in 2010 and before, and those published after the ACRM call for action. KT initially charted the studies, and MAO reviewed them to ensure accuracy. A final review was conducted by EvW.

### In step 5, we collated, summarized and reported the results

We worked as a team to synthetize the findings in an iterative manner. In particular, MAO and EvW took the responsibility to appraise the quality of the studies reviewed on the basis of the information on study population, study design, outcome assessment and analysis. KT and MAO then engaged in a series of interactive discussions about the relevance of the retained studies to the overarching research question and project goals. We used descriptive and frequency tables to aggregate the data, distinguishing the two publication time periods as previously noted, starting with the first year we could identify a relevant article, which was 1991.

## Results

**Fig. 1:**
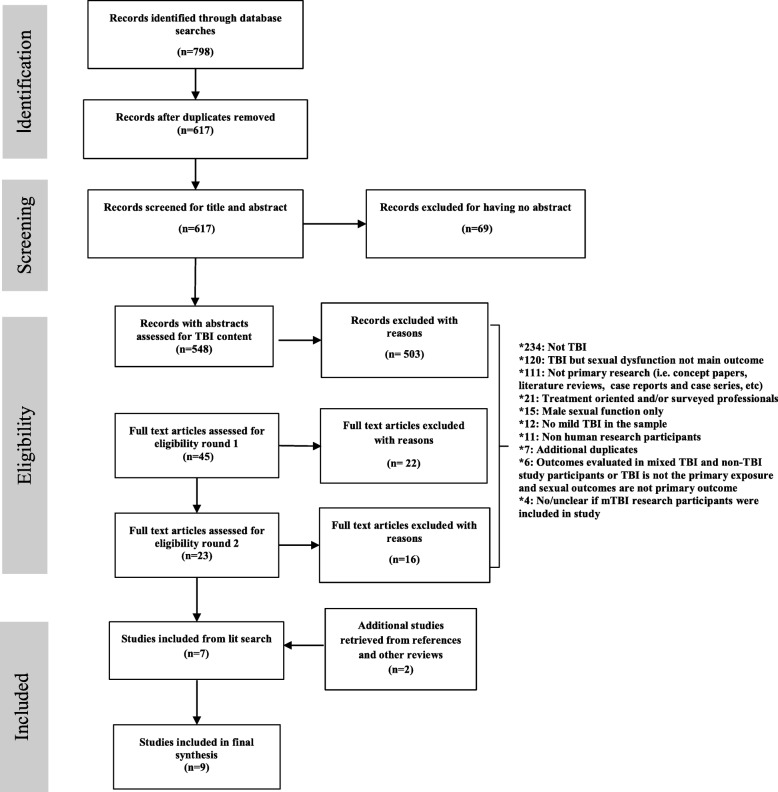
Flow chart of steps used in the selection of relevant studies

The initial search identified 798 records from the three databases (Fig. 1). After the librarian removed 181 duplicates, we had 617 records to review. Sixty nine were missing abstracts, and the abstracts of the remaining 548 were reviewed for TBI content. Another 503 were excluded, of which 234 were not related to TBI and 111 were not primary research. Forty-five full text articles were requested and reviewed in the first round of full text reviews, and 15 TBI studies that focused exclusively on sexual changes in males were excluded, along with 7 previously unidentified duplicates. After reviewing the 23 remaining studies in round 2 of the full-test review, we made 16 additional exclusions: 12 focused solely on severe and/or moderate TBI and 4 were unclear about the inclusion of a mildly injured group. We reviewed the references of the retained 7 studies, and identified and included two additional studies that had been excluded earlier because they were missing abstracts. A total of 9 studies were included in the final scoping review (Table 1).

**Table 1:** Summary of Studies that have included women and mTBI groups in evaluating TBI-associated changes in sexual functioning (1991–2019)

Authors, (Year, Country)	Characteristics of Study Participants	Gender Distribution of Study Participants with Head Injuries	TBI Severity and Patient Type	Study Design	Measure used to assess sexual dysfunction	Relevant Findings
Publications prior to 2010 ACRM proceedings (2010 and before)						
O’Carroll et al. (1991, UK) (O'Carroll et al. 1991)	36 with closed head injury (17 non-injured partners used as comparison group)	17% female (*n* = 6) 83% male (*n* = 30)	Mild to moderate (Mild:30.6%) Patients previously admitted for head injury mean age 35.63 (+/− 11.55 SD) years	Prospective Cohort Study, outcomes assessed on average 4.06 years post injury	Golombok Rust Inventory of Sexual Satisfaction (GRISS)	Overall Findings: 50% of head injured study participants (compared to 9% of partners fell in the ‘dysfunctional’ range for the GRISS Gender-Specific Findings: Patient outcomes were male-focused, and partner outcomes were female-focused. No evaluation of sexual dysfunctions in head injured female study participants and their male partners.
Kreuter et al. (1998, Sweden) (Kreuter et al. 1998)	92 with traumatic brain injury. No comparison group	29% Female (*n* = 27) 71% Male (*n* = 65)	Mild to Severe TBI, (% mild not indicated) Rehabilitation Patients. Median age 40 years, range 20–70 years	Prospective Cohort Study with outcomes assessed 1–20 years post-injury (median 9 years)	Sexual Interest and Satisfaction Scale	Overall Findings: For all respondents, 24% reported decreased and 23% non-existing frequency of intercourse. A quarter (26%) had no experience of sexual activity together with a partner after the injury. More than half of the 92 respondents (56%) reported dissatisfaction with the frequency of sexual activity Gender-Specific Findings: No female-specific changes were highlighted. Mainly focused on male study participants
Hibbard et al. (2000, USA) (Hibbard et al. 2000a)	322 with traumatic brain injury (264 non-injured comparison group)	40% Female (*n* = 129) 60% Male (*n* = 193)	Mild to Severe TBI, (Mild:37% of female participants, 18% of male participants) Community dwelling TBI patients between ages 16-64 years	Retrospective Cohort Study with outcomes assessed at 9.64 years (+/− 8.17SD) years for men, 9.30 (+/− 9.17 SD) years for women	Sexual interest and functioning components of the Quality of Life Interview (QOLI) questionnaire	Overall Findings: When contrasted to individuals without disability, individuals with TBI reported more frequent: (1) physiological difficulties influencing their energy for sex, sex drive, ability to initiate sexual activities and achieve orgasm (*p* < 0.001); (2) physical difficulties influencing body positioning, body movement and sensation (*p* < 0.001), and (3) body image difficulties influencing feelings of attractive and comfort with having a partner view one’s body during sexual activity (*p* < 0.001). Gender-Specific Findings: Women with TBI reported more frequent difficulties in sexual arousal, pain with sex, masturbation and vaginal lubrication (*p* < 0.001) than women without TBI. For women with TBI, an endocrine disorder and level depression combined were the most sensitive predictors of sexual difficulties.
Gaudet et al. (2001, USA) (Gaudet et al. 2001)	50 with traumatic brain injury (55 non-injured participants as comparison group)	48% female (*n* = 24) 52% male (*n* = 26)	Mild to Severe TBI, (% mild not indicated) TBI support group participants, between ages 17–78 years	Prospective Cohort Study, time since injury not provided	Questionnaires created by investigators	Overall Findings: TBI individuals have more negative feelings about themselves, their sexuality, and their relationships with others. Gender-Specific Findings: Female participants with TBI have more positive feelings about their sexuality than male participants with TBI (*p* = 0.044)l
Publications following 2010 ACRM proceedings (2011–2019)						
Goldin et al. (2014, USA) (Goldin et al. 2014)	220 with traumatic brain injury (83 non-injured participants as comparison group)	48% female (*n* = 105) 52% male (*n* = 115)	Mild to Severe TBI, (% mild not indicated) Community Dwelling individuals with average age 48 years (SD = 12.1)	Prospective Cohort Study, at least 12 months post-injury	Participation Objective Participation Subjective (POPS) questionnaire	Overall Findings: Individuals with TBI reported sex to be less important to their overall quality of life than comparison group without TBI. Gender-Specific Findings: Women with TBI reported having sex less frequently (*p* = 0.006) and rated sexual activity of less importance (*p* = 0.015) than did men with TBI. These sex differences were not observed in the comparison group (*p* = 0.421, *p* = 0.283, respectively).
Moreno et al. (2014, Colombia) (Moreno et al. 2014)	28 with traumatic brain injury (27 non-injured partner participants as comparison group)	32.1% female (*n* = 9) 67.9% male (*n* = 19)	Mild to Severe TBI, (% mild not indicated) University Hospital with participant average age of 39.7 years (SD = 11.46)	Prospective Cohort Study,on average 21.3 months post-injury (SD = 12.5).	Sexual Quality of Life Questionnaire (SQoL) Index of Sexual Satisfaction (ISS) Relationship Assessment Scale (RAS)	Overall Findings: Individuals with TBI scored significantly lower than healthy controls on the SQoL (*p* < 0.0001) and RAS (*p* < 0.0001) and had higher scores in the ISS (*p* < 0.0001). Gender-Specific Findings: None Results not stratitifed by sex/gender
Moreno et al., (2015, Canada) (Moreno et al. 2015a)	41 with traumatic brain injury (41 non-injured participants as comparison group)	56.1% female (*n* = 23) 43.9% male (*n* = 18)	Mild to Severe (65.9% mild) Rehabilitation centre in Montreal with participant average age of 38 years (SD = 9.8)	Prospective Cohort Study,on average 2.6 years post-injury (SD = 1.4).	Sexual Quality of Life Questionnaire (SQoL)	Overall Findings: Compared to healthy controls, individuals with TBI showed lower sexual quality of life, (t (73) = 2.5, *p* < 0.05) and was negatively and significantly correlated with the total Postconcussion Symptom Scale score (*r* = −0.69, *p* < 0.01) Gender-Specific Findings: None. Results not stratitifed by sex/gender
Downing & Ponsford (2018 (Australia) (Downing and Ponsford 2018)	55 with traumatic brain injury (55 non-injured partners participants as comparison)	25.5% female (*n* = 11) 74.5% males (*n* = 41)	Mild to Severe (27.3% mild) Rehabilitation programme at Epworth Healthcare with participant mean age of 34.6 years (SD = 12.8	Prospective Cohort Study,on average 36 months post-injury (SD = 3)	Derogatis Interview for Sexual Function-Self Report (DISF-SR)	Overall Findings: Participants with TBI obtained lower T-scores than partners on all subscales. Gender-Specific Findings: On the sexual arousal subscale, the difference between groups approached significance for females on lubrication subscale χ2(4, *N* = 50) = 9.46, *p* = .051. There were no other differences observed for any other items on the DISF-SR.
Anto-Ocrah et al. (2019, USA) (Anto-Ocrah et al. 2019a)	31 with traumatic brain injury (55 extremity injured comparison group)	100% female	Mild only (100% mild) Emergency Department of Level-One Trauma Center mean age 30.7 (SD = 8.2)	Prospective Cohort, with outcomes assessed between 6 and 10 weeks post concussion	Brain Injury Questionnaire on Sexuality (BIQS)	Overall & Gender-Specific Findings: Compared to extremity injury women, those with concussions had 1.70 increased risk of sexual dysfunction (RR1.70, 95% CI:1.04, 2.76; *p* = 0.03)

Ranging from publication dates of 1991 to 2019, the 9 articles (Table 1) included in this review were published by research teams from the America’s (USA (Anto-Ocrah et al. 2019a; Hibbard et al. 2000b; Gaudet et al. 2001; Goldin et al. 2014), Canada (Moreno et al. 2015a), Colombia (Moreno et al. 2014)), Europe (UK (O'Carroll et al. 1991), Sweden (Kreuter et al. 1998)), and Australia (Downing and Ponsford 2018). Four of the nine were published before the 2010 ACRM call for action (O’Carroll (1991) (O'Carroll et al. 1991), Kreuter (1998) (Kreuter et al. 1998), Hibbard (2000) (Hibbard et al. 2000a), Gaudet (2001) (Gaudet et al. 2001)) and the remaining 5 were published after the announcement (Goldin (2014) (Goldin et al. 2014), Moreno (2014) (Moreno et al. 2014), Moreno (2015) (Moreno et al. 2015a), Downing (2018) (Downing and Ponsford 2018), Anto-Ocrah (2019) (Anto-Ocrah et al. 2019a)). As depicted in Table 1, the representation of female research participants increased steadily over the 28-year timespan, ranging from 17% in 1991 to 100% by 2019. In the pre-2010 era, the trend is almost linear for the gender representation, beginning with O’Carroll’s 17% female study participants in 1991, 29% for the Kreuter study 7 years later, 40% in 2000 with Hibbard and team, and 48% female representation with Gaudet at al’s publication in 2001. The proportion of females is greater in the post-2010 time period, with the lowest representation being 32.1% in 2014 with Moreno and team’s publication, and ending with an all female study by Anto-Ocrah et al. in 2019. Paralleling this surge in gender representation is an increasing emphasis on mTBI research participants. Whereas they constituted no more than a third of study populations in the earlier 1991–2010 era, women with mTBI were 100% of the study sample in the 2019 Anto-Ocrah et al. study.

All four pre-2010 studies provided gender specific details on the effects of the head injury on sexual outcomes; though O’Carroll (1991) and Kreuter (1998) focused exclusively on male study participants and provided no insights on the sexual changes for the head injured women enrolled in their study. In contrast, Hibbard (2000) provided not only details on TBI and female sexuality, but elaborated on the sexual domains impacted, and the endocrinological and psychological predictors of the observed adverse outcomes. Gaudet followed suit in 2001, but with interestingly positive findings regarding the association between TBI and sexual dysfunction. Compared to male study participants, the team observed that females with TBI reported more positive feelings about their sexuality.

In the post-2010 period, two studies failed to provide gender-stratified findings (Moreno 2014 and 2015) while all others provided necessary details. Similar to Hibbard’s 2001 findings, Golding (2014) and Downing (2018) reported worse post-TBI sexual changes for women compared to men. Golding reported statistically significant differences in sexual frequency and the importance of sexuality for injured women compared to men, noting that these differences were not seen in the comparison groups. Downing reported on the effects of the injury on the lubrication sub-scale of the Derogatis Interview for Sexual Function-Self Report (DISF-SR). Comparing outcomes in all injured female research study participants, findings by Anto-Ocrah et al. (2019) echoed these earlier results and revealed that compared to extremity injured groups, women with mTBI have 70% greater risk of reporting sexual changes (RR1.70, 95% CI:1.04, 2.76; *p* = 0.03), though no evaluation of sexual domains was conducted.

All studies used cohort designs to assess outcomes across the two time periods, and other than Anto-Ocrah et al., who evaluated acute sexual changes (6-10 weeks post-injury), the remaining 8 studies focused on chronic changes (1 to 20 plus years after injury). Study participants were recruited from a mix of community, rehabilitation and hospital settings, and outcomes were assessed with a heterogeneous array of sexuality questionnaires, including the Brain Injury Questionnaire for Sexuality (BIQS) (Anto-Ocrah et al. 2019a; Ponsford et al. 2014; Stolwyk et al. 2013).

## Discussion

Human sexuality is a complex and multidimensional construct that includes the interaction of various biological, intra-and interpersonal and socio-cultural factors.There is an increasing interest in the role of sexuality in neurorehabilitation, and mounting awareness of the fact that many individuals with neurodisabilities experience problems with their sexual lives (Dyer and das Nair 2014; Moreno et al. 2017). This increased awareness has led to a better acknowledgment within the scientific community, of the importance of sexuality as a health outcome to promote the QOL of individuals with neurodisabilities; and the subsequent emergence of the field of neurosexuality; which seeks to better understand the relationship between the brain and sexuality in the context of neurodisabilities (Dyer and das Nair 2014; Moreno et al. 2017; Moreno et al. 2015b). Guided by the principles of neurosexuality, we sought to appraise the evidence base of how research teams have included injured females and concussion groups in evaluating TBI-related changes in sexual functioning. Our scoping review of the literature identified 9 publications between the search period of 1980 to 2019, that included women and mTBI study participants in the evaluation of sexual changes after TBI. Interestingly, the number of publications in the era before the ACRM call for action and afterwards were almost identical. But the proportion of women and individuals with mTBI included in the post-2010 era, was higher than the earlier time period. Over time, research groups seemed to be more conscientious about including more women in study populations, and provided gender-stratified results where appropriate. Consistently, head-injured women reported adverse sexual outcomes compared to head-injured males, orthopaedic, and non-injured comparison groups. This observation persisted regardless of recruitment site (rehabilitation center/ED/Community) or time of outcome assessment (acute versus chronic). The findings also remained despite the heterogeneity of survey questionnaires used to evaluate sexual functioning outcomes. It is unclear however, how these gender-specific differences vary by severity. Excluding the most recent 2019 study by Anto-Ocrah et al. (Anto-Ocrah et al. 2019a) which included all women, none of the research groups compared the findings by TBI severity, making it difficult to fully understand how concussion-related sexual changes compare to more severe forms of the head injury. Even though more mTBI study participants were included over the 28-year timespan of the review, comparison of sexual outcomes by TBI severity (mild vs moderate vs severe) was mostly lacking. Typical concussion symptoms resolve within weeks, but studies show that a “miserable minority” of those who sustain mTBIs experience symptoms that linger into months, if not years. These prolonged sequelae of outcomes often affects functional and vocational outcomes, and impact patients’ ability to resume their baseline functional status; including their ability to resume and sustain employment (Iaccarino 2018; De Koning et al. 2018; Meehan 3rd et al. 2016; Losoi et al. 2016). Because concussed patients are often discharged from the ED and not admitted, rigorous follow-up may be needed to ensure that these patients, females in particular, get adequate follow-up care to abate some of the long term consequences of the injury-sexual changes included. Providers should be trained to address sexual health after concussions with their patients in the outpatient setting, and prescribe the necessary behavioral and/or pharmacological therapies required to address the concerns raised by their head injured patients (Parish et al. 2019; Deschênes et al. 2019; Deschenes et al. 2017). This is especially crucial given the higher prevalence of pituitary dysfunctions in concussed (16.8%) compared to moderately injured TBI (10.9%) patients. (Schneider et al. 2007; West and Sharp 2014; Tanriverdi et al. 2010; Wagner et al. 2010)

Gonadotropin deficiencies, downstream effects of pituitary insults, have been associated with excess morbidity in affected patients (Schneider et al. 2007; West and Sharp 2014; Tanriverdi et al. 2010; Wagner et al. 2010). Decreased testosterone levels for example, could result in impaired cognitive function, resulting in the affected individuals being limited in their capacity to interpret external sexual stimuli into appropriate sexual response (Giacomo Ciocca et al. 2016; Cappelletti and Wallen 2016; Davis 2013; Roney and Simmons 2013). Depression and other mood disorders could ensue; either as a direct consequence of the hormonal deficiencies, or as a result of the physiological and physical changes imposed by the condition; impacting the individuals’ desire or ability to engage in sexual activity. These changes may inadvertently affect the person’s relationship status, in the long run, impair fertility outcomes (Parish et al. 2019; FertilityFactor.com 2016; Polyzos et al. 2016; Ascoli and Cavagnini 2006; Bell and Pepping 2001). Infertility, defined as the inability to conceive after one or more years of trying (American Society for Reproductive Medicine 2016), is a direct consequence of both hypogonadism and sexual dysfunctions (FertilityFactor.com 2016; Polyzos et al. 2016; Ascoli and Cavagnini 2006; Bell and Pepping 2001). The condition affects an estimated 13 million individuals in the United States alone (American Society for Reproductive Medicine 2016) and has been associated with high levels of major depression, anxiety and marital discord (McAnulty and Burnette 2006; American Society for Reproductive Medicine 2016; Centers for Disease Control and Prevention, Division of Reproductive Health n.d.). Despite the universal use of cohort designs to evaluate sexual health outcomes across the 9 studies included in this scoping review, none evaluated the impact of the dysfunctions on relationship status or parity.

A woman’s gender role in society as a wife, mother, and daughter could result in a much more differentiated constellation of family dynamics when TBI is introduced, than a man (Bell and Pepping 2001). The detrimental effects of the injury on expected gender norms may be even more salient in conservative non-western settings which are grossly under-represented in the literature. Not a single study included in this scoping review was conducted in developing settings in African or Asian regions, where motor vehicle crashes-the number one cause of TBIs (Cassidy et al. 2014; Anto-Ocrah et al. 2019a; Hartvigsen et al. 2014; Cassidy et al. 2004; Guerrero et al. 2000)-are most prevalent; and where sexual matters may be more taboo; making those with sexual dysfunctions more vulnerable to poorer quality of life (QOL) outcomes. Attention to these nuanced QOL measures (fertility, relationship status, gendered discussions of sexuality and sexual functioning, etc.) could shed more light on the longitudinal and “systems” impact of the head injury-beyond the patient.

The use of different sexual functioning scales across the 9 studies also presents a problem across the literature. Using scales like the Derogatis Interview for Sexual Function (DISF) (Downing and Ponsford 2018), the Golombok Rust Inventory of Sexual Satisfaction (GRISS) (O'Carroll et al. 1991), and other non-TBI specific measures do not allow the patient or the provider to evaluate relative changes in sexual functioning. These scales only provide cross-sectional assessments of the outcome, without references to pre-injury status. Temporal assessments are crucial to the evaluation of sexual changes to avoid the possibility of over or under-diagnosing the dysfunction. Scales like the Brain Injury Questionnaire on Sexuality (BIQS), which is designed to account for temporal changes in sexual function, is accommodating of a variety of control groups, and is not restricted to married and/or heterosexual individuals, is one of the few questionnaires designed to assess sexual outcomes in individuals with brain injuries (Latella et al. 2018; Stolwyk et al. 2013; Ponsford et al. 2013). The BIQS, which was used by only one (Anto-Ocrah et al. 2019b) of the 9 studies included in the review, should be used more consistently to allow for comparisons of neurosexual outcomes across multiple studies in the literature.

One of the limitations of our review is that not all injuries studied across the 9 included studies were mTBI/concussions. Thus using these mixed-severity studies to address the outcomes of mTBI/concussions exclusively, could be potentially biased.

In order to tailor interventions for the appropriate group of TBI patients, cohort studies and other epidemiologic research in neurosexuality are needed to better understand the effects of concussions, the most common form of TBI on the sexual health of women, one of the most rapidly growing and vulnerable subgroups within the head injured population. The long term effects of the sexual changes, such as infertility and relationship discord should also be evaluated to better understand the longitudinal impact of the injury. Consideration should be given to the sexuality of lesbian and transgender women with mTBI, the sexuality of older women with mTBI, and other such under-represented subgroups within the TBI population, who are often over-looked across the literature. Research funds are needed to overcome some of the unique challenges associated with engaging such under-represented groups in research, in order to advance the field. Fiscal and logistical limitations however, should not deter the scientific community from actively engaging women and other under-represented populations in such epidemiological endeavors. Such research is necessary to inform policy and advocate for the allocation of fiscal resources to further train rehabilitation professionals, neuroscientists, sex therapists and other mental health providers, to further integrate neurosexual outcomes for the diverse spectrum of patients who may be seeking neuro-rehabilitative care.

## Conclusions

Women with TBI have been historically under-presented in clinical research. Compared to the sexuality of women with neurodisabilities, male sexual problems and treatments are better documented (Latella et al. 2018; Bell and Pepping 2001). Although this is not entirely surprising in light of the preponderance of men with TBI, it also reflects the traditional tendency of medical researchers to concentrate their efforts on the male form as the “norm” (Beery and Zucker 2011; National Institutes of Health 2018; Bell and Pepping 2001). In our scoping review of the literature, we found that the number of publications in the era before the ACRM call for action and afterwards were almost identical. The representation of female research as well as individuals with mTBI increased steadily over the 28-year timespan. The introduction of the National Institutes of Health’s’ “sex as a biological variable” (SABV) requirement in clinical research will hopefully continue to provide the impetus needed to improve the attention paid to TBI-related neurosexual outcomes in women, particularly those with concussions, who are greater, both in numbers and undiagnosed outcomes.

## Data Availability

The datasets used and/or analyzed during the current study are available from the corresponding author on reasonable request.

## Appendix

### Search Terms

#### PubMed

(Sexuality OR Sexual Dysfunction, Physiological OR Sexual Dysfunctions, Psychological OR Sexual Behavior OR Sexual Health) AND (brain concussion OR Head Injuries, Closed OR Brain Injuries, Traumatic OR Brain Concussion OR Concussion)

#### Embase

(‘sexuality’/exp. OR ‘psychosexuality’ OR ‘sexual functioning’ OR ‘sexual habit’ OR ‘sexual hygiene’ OR ‘sexual partners’ OR ‘sexual reinforcement’ OR ‘sexual relation’ OR ‘sexuality’ OR ‘sexual dysfunction’/exp. OR ‘dysfunction, sexual’ OR ‘sex abnormality’ OR ‘sex disorders’ OR ‘sex dysfunction’ OR ‘sex insufficiency’ OR ‘sex problem’ OR ‘sexual and gender disorders’ OR ‘sexual asthenia’ OR ‘sexual disability’ OR ‘sexual disorder’ OR ‘sexual disturbance’ OR ‘sexual dysfunction’ OR ‘sexual dysfunction, physiological’ OR ‘sexual problem’ OR ‘sexual behavior’/exp. OR ‘behavior, reproductive’ OR ‘behavior, sexual’ OR ‘behaviour, reproductive’ OR ‘behaviour, sexual’ OR ‘sex behavior’ OR ‘sex behaviour’ OR ‘sex life’ OR ‘sex, extramarital’ OR ‘sexual action’ OR ‘sexual activity’ OR ‘sexual behavior’ OR ‘sexual behaviour’ OR ‘sexual behaviour, animal’ OR ‘sexual life’ OR ‘sexual receptivity’ OR ‘sexual response’ OR ‘sexual health’/exp. OR ‘sexual health’) AND (‘concussion’/exp. OR ‘traumatic brain injury’/exp. OR ‘brain injuries, traumatic’ OR ‘brain lesion, traumatic’ OR ‘brain system trauma’ OR ‘brain trauma’ OR ‘cerebral trauma’ OR ‘cerebrovascular trauma’ OR ‘encephalopathy, traumatic’ OR ‘mild traumatic brain injury’ OR ‘organic cerebral trauma’ OR ‘posttraumatic encephalopathy’ OR ‘traumatic brain injuries’ OR ‘traumatic brain injury’ OR ‘traumatic brain lesion’ OR ‘traumatic cerebral lesion’ OR ‘traumatic encephalopathy’) AND english:la

#### Web of Science

TS = (Sexuality OR Sexual Dysfunction OR Sexual Behavior OR Sexual Health) AND TS = (concussion OR traumatic brain injury).

## Abbreviations

ACRM: American Congress of Rehabilitation Medicine
BIQS: Brain Injury Questionnaire for Sexuality
CDC: Centers for Disease Control and Prevention
DISF-SR: Derogatis Interview for Sexual Function-Self Report
ED: Emergency Department
FDA: Food and Drug Administration
mTBI: Mild traumatic brain Injury
NIH: National Institutes of Health
QOL: Quality of life
TBI: Traumatic brain injury
WHO: World Health Organization
